# Supplementary material for: Network-based modelling reveals cell-type enriched patterns of non-coding RNA regulation during human skeletal muscle remodelling
Source: NAR Mol Med. 2024 Oct 22;1(4):ugae016. doi: 10.1093/narmme/ugae016 (PMC11632610; doi:10.1093/narmme/ugae016)
Supplement: ugae016_Supplemental_Files [file ugae016_Supplemental_Files.zip › Supplementary Data_WithReferences.docx]

**SUPPLEMENTAL MATERIALS**

**Title:** Network-based modelling reveals cell-type enriched patterns of non-coding RNA regulation during human skeletal muscle remodelling

**Authors:** Jonathan C. Mcleod ^1^, Changhyun Lim^1,2^, Tanner Stokes^1^, Jalil-Ahmad Sharif ^3^, Vagif Zeynalli ^1^, Lucas Wiens ^1^, Alysha C D’Souza ^1^, Lauren Colenso-Semple ^1^, James McKendry ^1,4^, Robert W. Morton ^1^, Cameron J. Mitchell ^5^, Sara Y. Oikawa ^1^, Claes Wahlestedt ^6^, J Paul Chapple ^3^, Chris McGlory ^7^, James A. Timmons ^3,6*^ and Stuart M. Phillips ^1*^

**Short-title:** RNA profiling of ncRNA genes in skeletal muscle

**One Sentence Summary:** We used an optimised transcriptomic strategy to identify a set of ncRNA genes regulated during skeletal muscle hypertrophy in one hundred and forty-four people, with network modelling and spatial imaging providing biological context.

**Key words:** Transcriptome, Angiogenesis, Spatial, Muscle Growth, Exercise, Single-Cell, Immune Cell

**Affiliations:**

^1^Department of Kinesiology, McMaster University, Hamilton, Ontario, Canada.

^2^ Population Health Sciences Institute, Faculty of Medical Sciences, Newcastle University, Newcastle upon Tyne, UK

^3^ Faculty of Medicine and Dentistry, Queen Mary University London, London, UK.

^4^ Faculty of Land and Food Systems, Food, Nutrition & Health, University of British Columbia, BC, Canada

^5^ School of Kinesiology, University of British Columbia, BC, Canada

^6^ University of Miami Miller School of Medicine, Miami, FL, USA

^7^ School of Kinesiology and Health Studies, Queens University, Kingston, ON, Canada

* Joint senior authors

Correspondence to: Jonathan C. Mcleod, Jonathan.mcleod@queensu.ca

**Table S1.** Demographics of the five studies used for establishing ncRNA genes associated with skeletal muscle hypertrophy.

|  | Morton et al., 2019 (1) | Morton et al., 2016 (2) | Phillips et al., 2017 (3) | Mitchell et al., 2014 (4) | Stokes et al., 2020 (5) |
| --- | --- | --- | --- | --- | --- |
| Sample size, n | 32 | 33 | 47 | 20 | 12 |
| Age, years | 22 ± 3 (19 – 28) | 23 ± 3 (20 – 29) | 38 ± 9 (21 – 51) | 24 ± 3 (20 – 30) | 21 ± 3 (18 – 29) |
| Gender, M/F | 32/0 | 33/0 | 18/29 | 20/0 | 12/0 |
| Body mass index, kg/m^2^ | 25 ± 6 (18 – 38) | 26 ± 9 (41) | 32 ± 4 (26 – 43) | 24 ± 4 (15 – 32) | 24 ± 3 (20 – 31) |
| Measurement instrument | DXA | DXA | DXA | MRI | DXA |
| Limbs Involved in Measurement | 1 | 2 | 1 | 1 | 1 |
| Pre-training LLM, kg | 10.3 ± 2.1 (7.2 – 14.6) | 24.2 ± 3.0 (17.4 – 30.9) | 5.8 ± 1.4 (3.2 – 8.6) | - | 9.5 ± 1.6 (7.7 – 13.4) |
| Post-training LLM, kg | 10.6 ± 2.1 (7.6 – 15.2) | 25.0 ± 3.0 (19.9 – 31.2) | 6.0 ± 1.4 (3.5 – 8.7) | - | 9.9 ± 1.6 (8.1 – 13.4) |
| Pre-training QMV, cm^3^ | - | - | - | 1862.0 ± 402.6 (1039.0 – 2822.0) | - |
| Post-training QMV, cm^3^ | - | - | - | 1985.0 ± 417.6 (1278.0 – 3104.0) | - |
| dLLM, % | 3.2 ± 3.1 (-3.7 – 9.1) | 3.0 ± 5.0 (-5.0 – 19.0) | 3.1 ± 3.7 (-4.5 – 11.0) | - | 5.0 ± 4.3 (-1.2 – 14.0) |
| dQMV, % | - | - | - | 7.1 ± 7.0 (-1.8 – 24.7) | - |
| *R*^2^ dLLM (kg) vs Pre-training LLM (kg) | 0.07 | 0.01 | < 0.01 | - | < 0.01 |
| *R*^2^ dQMV (cm^3^) vs Pre-training QMV (cm^3^) | - | - | - | < 0.01 | - |
| *R*^2^ dLLM (kg) vs Age (years) | 0.26 | < 0.01 | < 0.01 | - | 0.04 |
| *R*^2^ dQMV (cm^3^) vs Age (years) | - | - | - | 0.01 | - |
| *R*^2^ dLLM (kg) vs Gender | - | - | 0.04 | - | - |
| LMR group, n | 20 | 20 | 24 | 14 | 10 |
| NMLR group, n | 10 | 13 | 20 | 5 | 2 |

Age, body mass index, Pre-training LLM, Post-training LLM, dLLM, Pre-training QMV, Post-training QMV, and dQMV are displayed as mean ± SD (min - max). Sample size, Gender, LMR group, and NMLR group are displayed as counts. Abbreviations: DXA, dual X-ray absorptiometry; MRI, magnetic resonance imaging; dLLM, delta leg lean mass; QMV, quadriceps muscle volume; LMR, lean mass responders; NMLR, no measurable lean mass response.

**
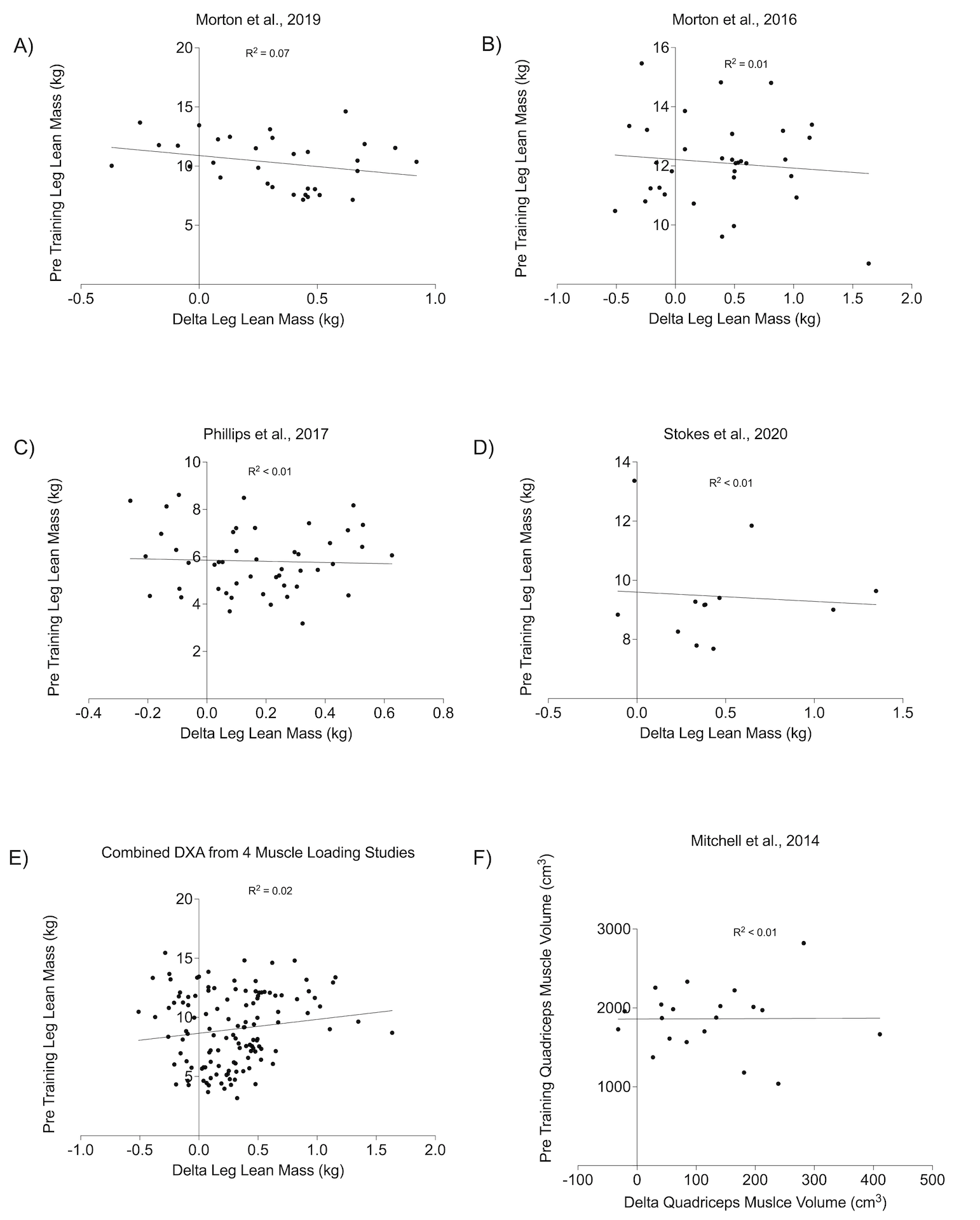
**

**Figure S1.** (A-D) Changes in leg lean mass versus pretraining leg lean mass for 4 muscle loading studies, and (E) depicts the aggregated relationship. F) Changes in quadriceps muscle volume vs pre-training quadriceps muscle volume.

**
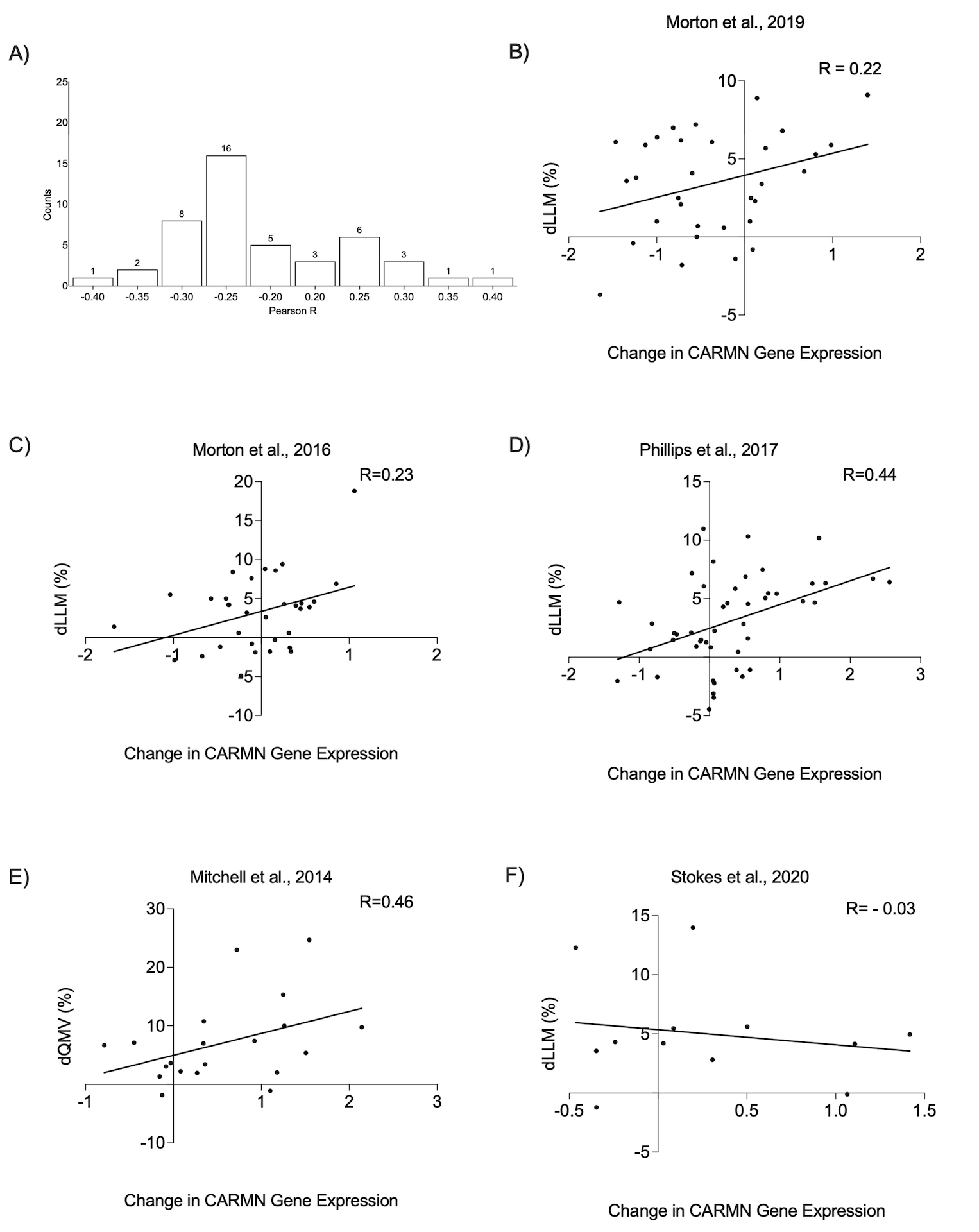
**

**Figure S2.** (A) Distribution of Pearson correlation coefficients among the 46 ncRNA genes containing a change in expression that was modestly associated with changes in LLM (dLLM), or changes in Quadriceps muscle volume (dQMV; Supplement Data S5). (B -F) Visual example from the linear modelling analysis, depicting the relationship between dLLM (or dQMV [E]) and changes in *CARMN* gene expression for each of the 5 individual exercise studies.

**
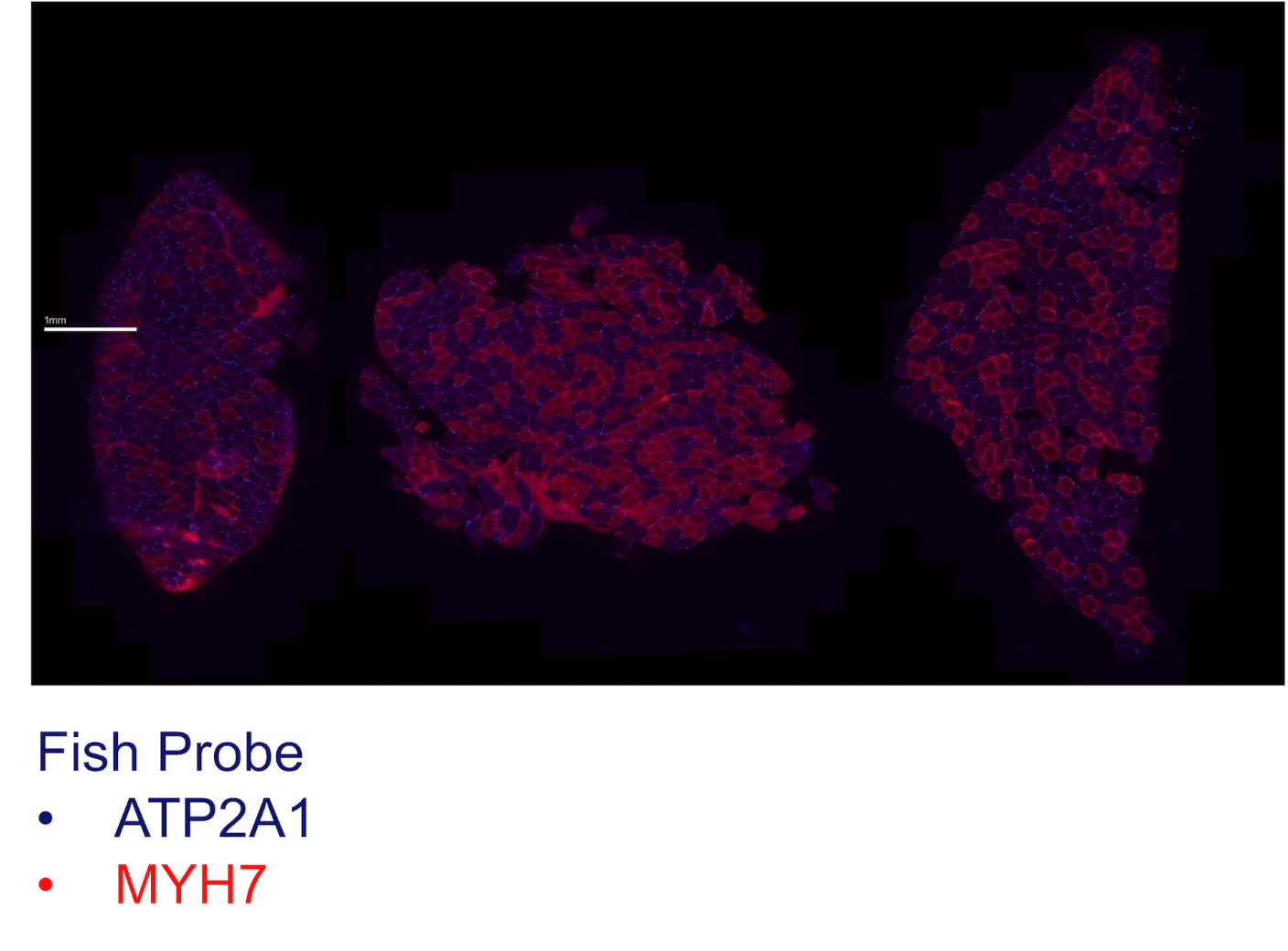
**

**Figure S3.** MERSCOPE-MERFISH overview for 3 samples used.

**Table S2.** Existing biochemical relationship for 110 hypertrophy-related ncRNA genes in skeletal muscle.

| **ENSG** | **Gene Symbol** | **Gene Synonyms** | **Group Identified In** | **Pre-existing evidence in skeletal muscle physiology** |
| --- | --- | --- | --- | --- |
| ENSG00000222041 | CYTOR | C2ORF59, LINC00152, MGC4677, NCRNA00152 | LMR | Exercise-induced lncRNA that is reduced with ageing. Promotes fast twitch cell fate. Reduces chromatin accessibility and sequesters TEAD1 (6). |
| ENSG00000268518 | ENSG00000268518 | MYREM, LNCFAM, LNCFAM71E1-2:2 | LMR | Increases during myogenesis and promotes differentiation of myoblasts into myotubes (7). Increases transcription of myosin binding protein C2 (7). |
| ENSG00000214548 | MEG3 | GTL2, LINC00023, NCRNA00023, ONCO-LNCRNA-83 | LMR | MEG3 regulates myoblast differentiation (8). MEG3 knockdown enhances mesenchymal characteristics, impairs myotube formation, and compromises skeletal muscle regeneration (8). |
| ENSG00000130600 | H19 | ASM, ASM1, D11S813E, LINC00008, MIR675HG, NCRNA00008 | LMR | Predictor VO2max training response (9). H19-encoded miRNA regulates skeletal muscle growth and translation initiation factors (10). Maintains slow muscle fibre phenotype, exercise endurance and muscle function in mice (11). In mice myoblasts, H19 associates with Dystrophin and protects its degradation (12). |
| ENSG00000241158 | ADAMTS9-AS1 | NONE | dLLM vs dGE | NONE |
| ENSG00000262943 | ALOX12P2_ENST00000570921 | NONE | LMR | NONE |
| ENSG00000215811 | BTNL10P | BTN4, BTNL10, BUTR1 | dLLM vs dGE | NONE |
| ENSG00000254614 | CAPN1-AS1 | NONE | LMR | NONE |
| ENSG00000249669 | CARMN | CARMEN, MIR143HG | LMR & dLLM vs dGE | NONE |
| ENSG00000272168 | CASC15 | LINC00340, LNC-SOX4-1 | dLLM vs dGE | NONE |
| ENSG00000229140 | CCDC26 | MGC27434, RAM | dLLM vs dGE | NONE |
| ENSG00000234898 | CHEK2P3 | NONE | LMR | NONE |
| ENSG00000229452 | CPVL-AS1 | NONE | dLLM vs dGE | NONE |
| ENSG00000226950 | DANCR | AGU2, ANCR, KIAA0114, LNCRNA-ANCR, SNHG13 | LMR | NONE |
| ENSG00000233143 | DIRC3-AS1 | NONE | LMR | NONE |
| ENSG00000232010 | DNMT3L-AS1 | NONE | LMR | NONE |
| ENSG00000223668 | EEF1A1P24 | NONE | LMR | NONE |
| ENSG00000149656 | ENSG00000149656 | NONE | NMLMR | NONE |
| ENSG00000176349 | ENSG00000176349 | NONE | dLLM vs dGE | NONE |
| ENSG00000213963 | ENSG00000213963 | NONE | NMLMR | NONE |
| ENSG00000214942 | ENSG00000214942 | NONE | LMR | NONE |
| ENSG00000228778 | ENSG00000228778 | NONE | dLLM vs dGE | NONE |
| ENSG00000229425 | ENSG00000229425 | NONE | LMR | NONE |
| ENSG00000230947 | ENSG00000230947 | NONE | dLLM vs dGE | NONE |
| ENSG00000231927 | ENSG00000231927 | NONE | dLLM vs dGE | NONE |
| ENSG00000234139 | ENSG00000234139 | NONE | dLLM vs dGE | NONE |
| ENSG00000234677 | ENSG00000234677 | NONE | dLLM vs dGE | NONE |
| ENSG00000236234 | ENSG00000236234 | NONE | dLLM vs dGE | NONE |
| ENSG00000236601 | ENSG00000236601 | NONE | LMR | NONE |
| ENSG00000237035 | ENSG00000237035 | NONE | dLLM vs dGE | NONE |
| ENSG00000238142 | ENSG00000238142 | NONE | NMLMR | NONE |
| ENSG00000249021 | ENSG00000249021 | NONE | LMR | NONE |
| ENSG00000250978 | ENSG00000250978 | NONE | LMR | NONE |
| ENSG00000251511 | ENSG00000251511 | NONE | dLLM vs dGE | NONE |
| ENSG00000252230 | ENSG00000252230 | NONE | NMLMR | NONE |
| ENSG00000253553 | ENSG00000253553 | NONE | dLLM vs dGE | NONE |
| ENSG00000254409 | ENSG00000254409 | NONE | NMLMR | NONE |
| ENSG00000254641 | ENSG00000254641 | NONE | LMR | NONE |
| ENSG00000254975 | ENSG00000254975 | NONE | dLLM vs dGE | NONE |
| ENSG00000255313 | ENSG00000255313 | NONE | dLLM vs dGE | NONE |
| ENSG00000255689 | ENSG00000255689 | NONE | LMR | NONE |
| ENSG00000258649 | ENSG00000258649 | NONE | dLLM vs dGE | NONE |
| ENSG00000258760 | ENSG00000258760 | NONE | NMLMR | NONE |
| ENSG00000260971 | ENSG00000260971 | NONE | NMLMR | NONE |
| ENSG00000261327 | ENSG00000261327 | NONE | dLLM vs dGE | NONE |
| ENSG00000263618 | ENSG00000263618 | NONE | LMR | NONE |
| ENSG00000267784 | ENSG00000267784 | NONE | LMR | NONE |
| ENSG00000273674 | ENSG00000273674 | NONE | dLLM vs dGE | NONE |
| ENSG00000279482 | ENSG00000279482 | NONE | LMR | NONE |
| ENSG00000285945 | ENSG00000285945 | NONE | LMR | NONE |
| ENSG00000286829 | ENSG00000286829 | NONE | LMR | NONE |
| ENSG00000288253 | ENSG00000288253 | NONE | dLLM vs dGE | NONE |
| ENSG00000289228 | ENSG00000289228 | NONE | LMR | NONE |
| ENSG00000290482 | ENSG00000290482 | NONE | dLLM vs dGE | NONE |
| ENSG00000291041 | ENSG00000291041 | NONE | LMR | NONE |
| ENSG00000273100 | ENST00000610240 | NONE | LMR | NONE |
| ENSG00000275563 | ENST00000613990 | NONE | LMR | NONE |
| ENSG00000260528 | FAM157C | NONE | NMLMR | NONE |
| ENSG00000251402 | FAM90A25P | NONE | LMR | NONE |
| ENSG00000256943 | GALNT9-AS1 | NONE | dLLM vs dGE | NONE |
| ENSG00000224934 | GOT1-DT | NONE | LMR | NONE |
| ENSG00000179362 | HMGN2P46 | C15ORF21, D-PCA-2, FLJ39426 | dLLM vs dGE | NONE |
| ENSG00000224699 | LAMTOR5-AS1 | NONE | dLLM vs dGE | NONE |
| ENSG00000230710 | LINC00332 | NCRNA00332 | LMR | NONE |
| ENSG00000226519 | LINC00390 | TCONS_00021641 | LMR | NONE |
| ENSG00000236678 | LINC00347 | NONE | LMR | NONE |
| ENSG00000225179 | LINC00457 | NONE | dLLM vs dGE | NONE |
| ENSG00000233723 | LINC01122 | AC007092.1, FLJ30838 | dLLM vs dGE | NONE |
| ENSG00000233985 | LINC01681 | NONE | dLLM vs dGE | NONE |
| ENSG00000226983 | LINC01692 | NONE | dLLM vs dGE | NONE |
| ENSG00000237166 | LINC01792 | NONE | dLLM vs dGE | NONE |
| ENSG00000267057 | LINC01905 | NONE | LMR | NONE |
| ENSG00000204650 | LINC02210 | C17ORF69, C17orf69, CRHR1-IT1, FLJ25168 | NMLMR | NONE |
| ENSG00000229536 | LINC02572 | AC079776.2 | NMLMR | NONE |
| ENSG00000233593 | LINC02609 | NONE | dLLM vs dGE | NONE |
| ENSG00000234222 | LIX1L-AS1 | NONE | LMR | NONE |
| ENSG00000267023 | LRRC37A16P | NONE | LMR | NONE |
| ENSG00000254349 | MIR2052HG | NONE | NMLMR | NONE |
| ENSG00000172965 | MIR4435-2HG | AGD2, AK001796, LINC00978, LNCRNA-AWPPH, MIR4435-1HG, MORRBID | LMR | NONE |
| ENSG00000197182 | MIRLET7BHG | LINC-PPARA | dLLM vs dGE | NONE |
| ENSG00000238151 | MLLT10P1 | BA348I14.3, MLLT10L | LMR | NONE |
| ENSG00000242086 | MUC20-OT1 | NONE | LMR | NONE |
| ENSG00000214106 | PAXIP1-AS2 | PAXIP1OS | dLLM vs dGE | NONE |
| ENSG00000229941 | PDE11A-AS1 | NONE | dLLM vs dGE | NONE |
| ENSG00000249996 | PPIC-AS1 | NONE | dLLM vs dGE | NONE |
| ENSG00000226833 | PPP1CB-DT | NONE | NMLMR | NONE |
| ENSG00000214182 | PTMAP5 | NONE | LMR | NONE |
| ENSG00000201875 | RN7SKP178 | NONE | LMR | NONE |
| ENSG00000275776 | RN7SL185P | NONE | LMR | NONE |
| ENSG00000239468 | RN7SL569P | NONE | dLLM vs dGE | NONE |
| ENSG00000239899 | RN7SL674P | NONE | NMLMR | NONE |
| ENSG00000252957 | RNA5SP402 | NONE | dLLM vs dGE | NONE |
| ENSG00000201474 | RNU6-164P | NONE | LMR | NONE |
| ENSG00000253084 | RNU6-840P | NONE | LMR | NONE |
| ENSG00000199260 | RNU6-874P | NONE | dLLM vs dGE | NONE |
| ENSG00000206732 | RNU6-936P | NONE | dLLM vs dGE | NONE |
| ENSG00000230438 | SERPINB9P1 | MGC39372 | LMR | NONE |
| ENSG00000234899 | SOX9-AS1 | NONE | LMR | NONE |
| ENSG00000187653 | TMSB4XP8 | TMSL3 | LMR | NONE |
| ENSG00000232600 | TONSL-AS1 | NONE | dLLM vs dGE | NONE |
| ENSG00000237298 | TTN-AS1 | NONE | LMR | NONE |
| ENSG00000273249 | WDR5-DT | NONE | NMLMR | NONE |
| ENSG00000199332 | Y_RNA_ENST00000362462 | NONE | NMLMR | NONE |
| ENSG00000199832 | Y_RNA_ENST00000362962 | NONE | LMR | NONE |
| ENSG00000212556 | Y_RNA_ENST00000391254 | NONE | dLLM vs dGE | NONE |
| ENSG00000252759 | Y_RNA_ENST00000516950 | NONE | dLLM vs dGE | NONE |
| ENSG00000252915 | Y_RNA_ENST00000517106 | NONE | dLLM vs dGE | NONE |
| ENSG00000250802 | ZBED3-AS1 | LNC13728 | dLLM vs dGE | NONE |
| ENSG00000263072 | ZNF213-AS1 | NONE | dLLM vs dGE | NONE |
| ENSG00000257267 | ZNF271P | HZF7, ZNF271, ZNFEB | LMR | NONE |

A systematic PUBMED search was carried out on March 19^th^, 2024, using the gene symbol (and using any related gene synonyms found on Ensembl (<https://useast.ensembl.org/index.html>), and the following terms: “skeletal muscle” AND “myotubes” AND “myocytes”. Relevant articles were examined to determine a clear role in skeletal muscle physiology.

**
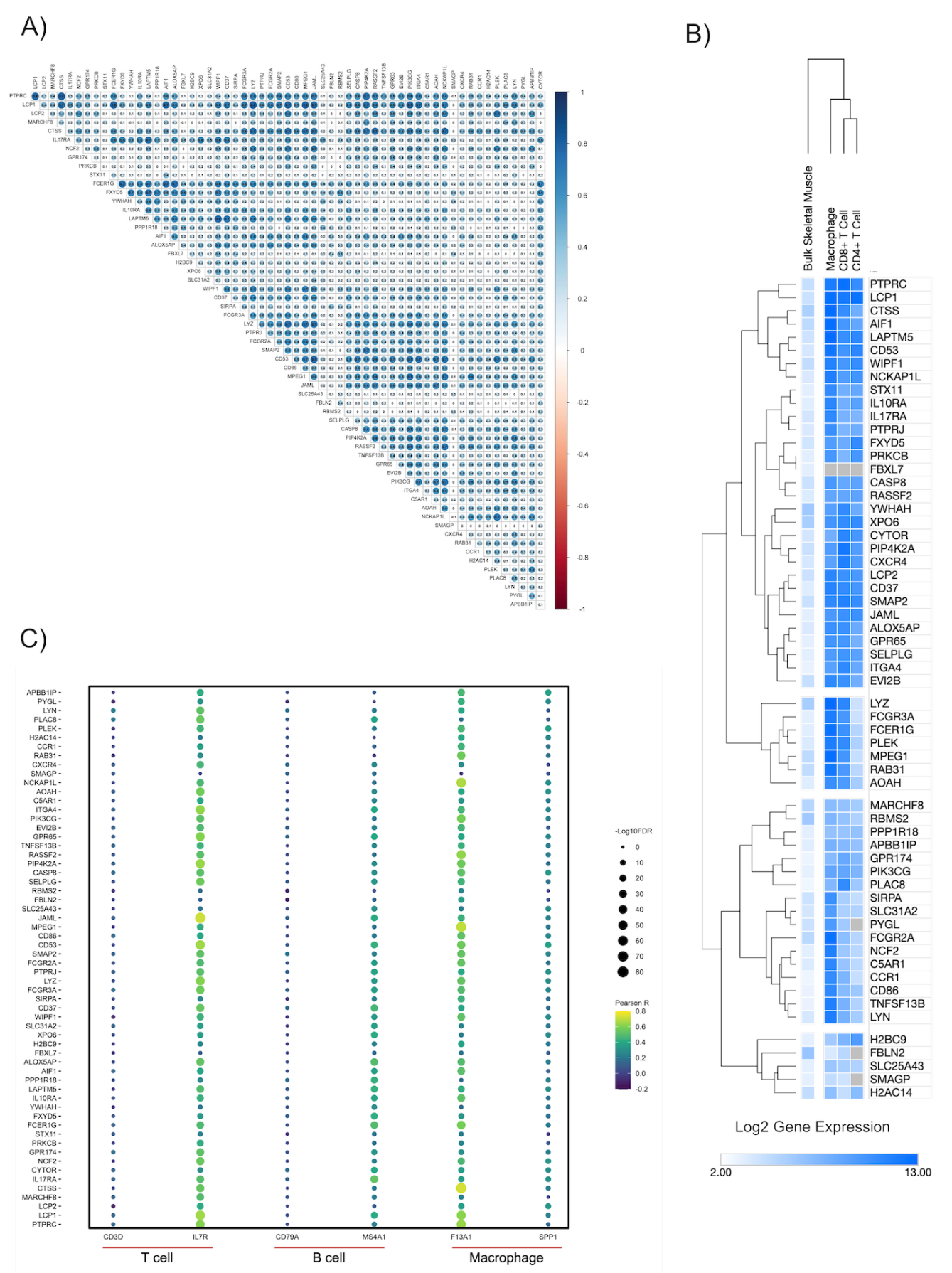
**

**Figure S4.** A) Pearson correlation matrix of all 60 genes co-expressed in network 1. Majority of the genes are positively correlated with each other in this network. B) the heatmap uses the log2 gene expression in skeletal muscle (n=437) and plots the 60 genes co-expressed in network 1 along with marker genes from three mononuclear cells of the immune system (macrophages, CD4 T-cells, and CD8 T-cells). The plot was created using Morpheus (https://clue.io/Morpheus), and genes and tissue types were hierarchically clustered using Euclidean distance (linkage method: complete). C) Dot plot depicting the association between the expression of network 1 genes, and gene markers for T-cells, B-cells and macrophages, in human skeletal muscle transcriptomic data (n=437). The colouring of the dot corresponds to the Pearson correlation coefficient, and the size of the dot is proportional to the –Log^10^ FDR.


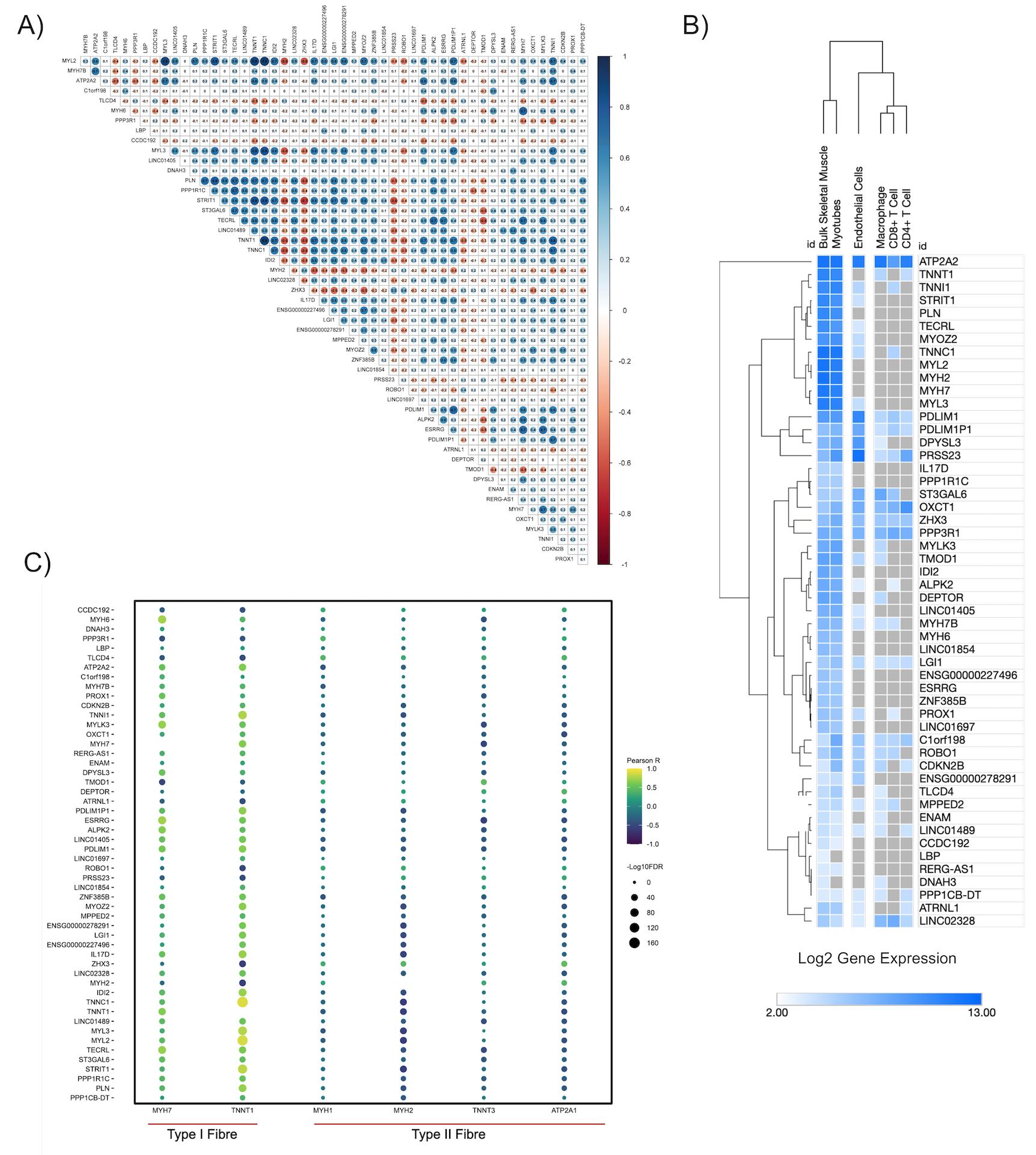


**Figure S5.** A) Pearson correlation matrix of all 52 genes co-expressed in network 2. Majority of the genes are positively correlated with each other in this network. b) the heatmap uses the log2 gene expression in skeletal muscle (n=437) and plots the 52 genes co-expressed in network 2 along with marker genes from bulk skeletal muscle, myotubes, endothelial cells, and three mononuclear cells of the immune system (macrophages, CD4 T-cells, and CD8 T-cells). The plot was created using Morpheus (https://clue.io/Morpheus), and genes and tissue types were hierarchically clustered using Euclidean distance (linkage method: complete). C) Dot plot depicting the association between the expression of network 2 genes, and gene markers for type I and type II fiber in human skeletal muscle transcriptomic data (n=437). The colouring of the dot corresponds to the Pearson correlation coefficient, and the size of the dot is proportional to the –Log^10^ FDR.

**
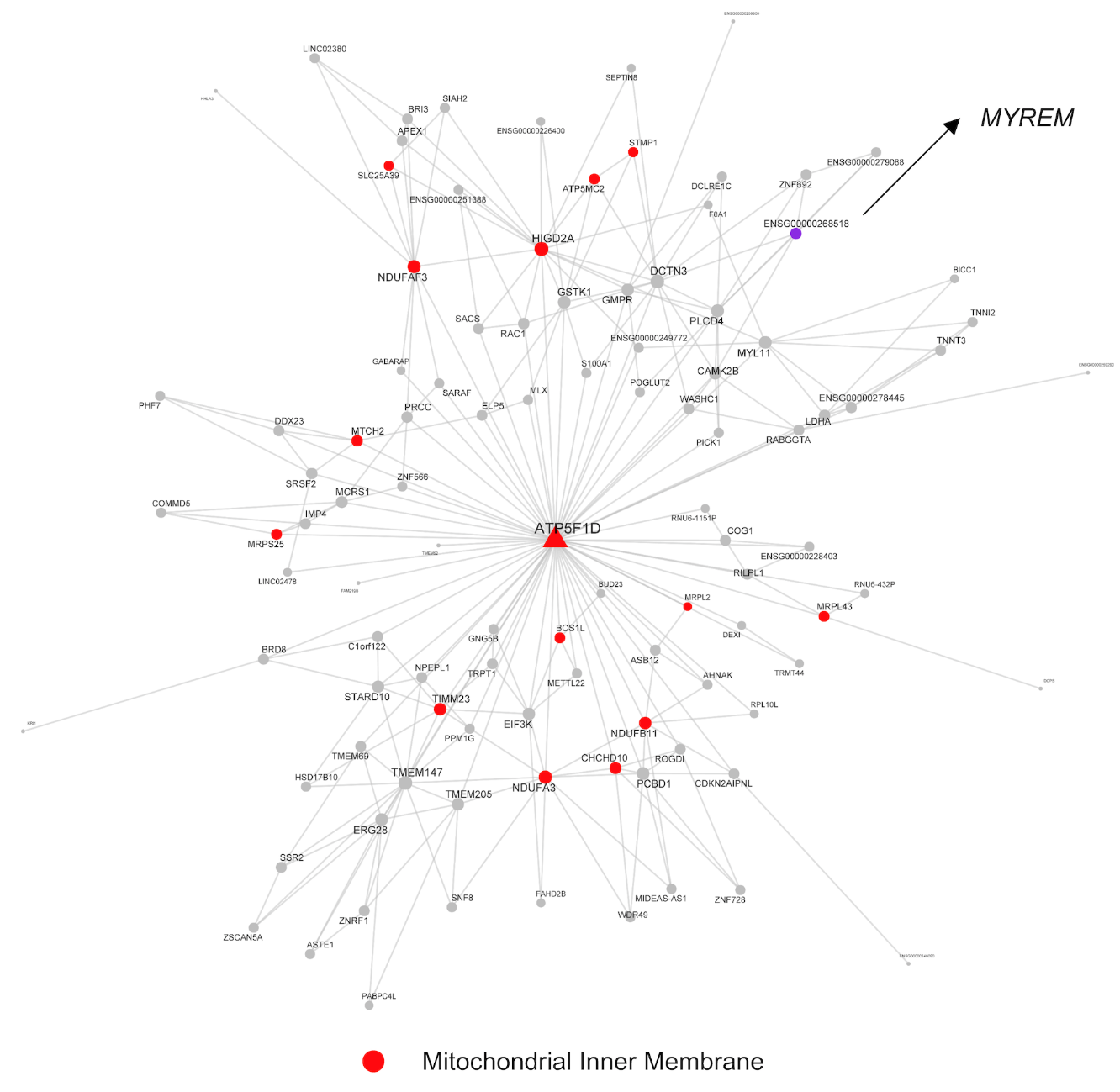
**

**Figure S6.** A mitochondrial-related gene co-expression network (network 4; Table 1) that contains the hypertrophy-related ncRNA gene, *MYREM* (purple).

**
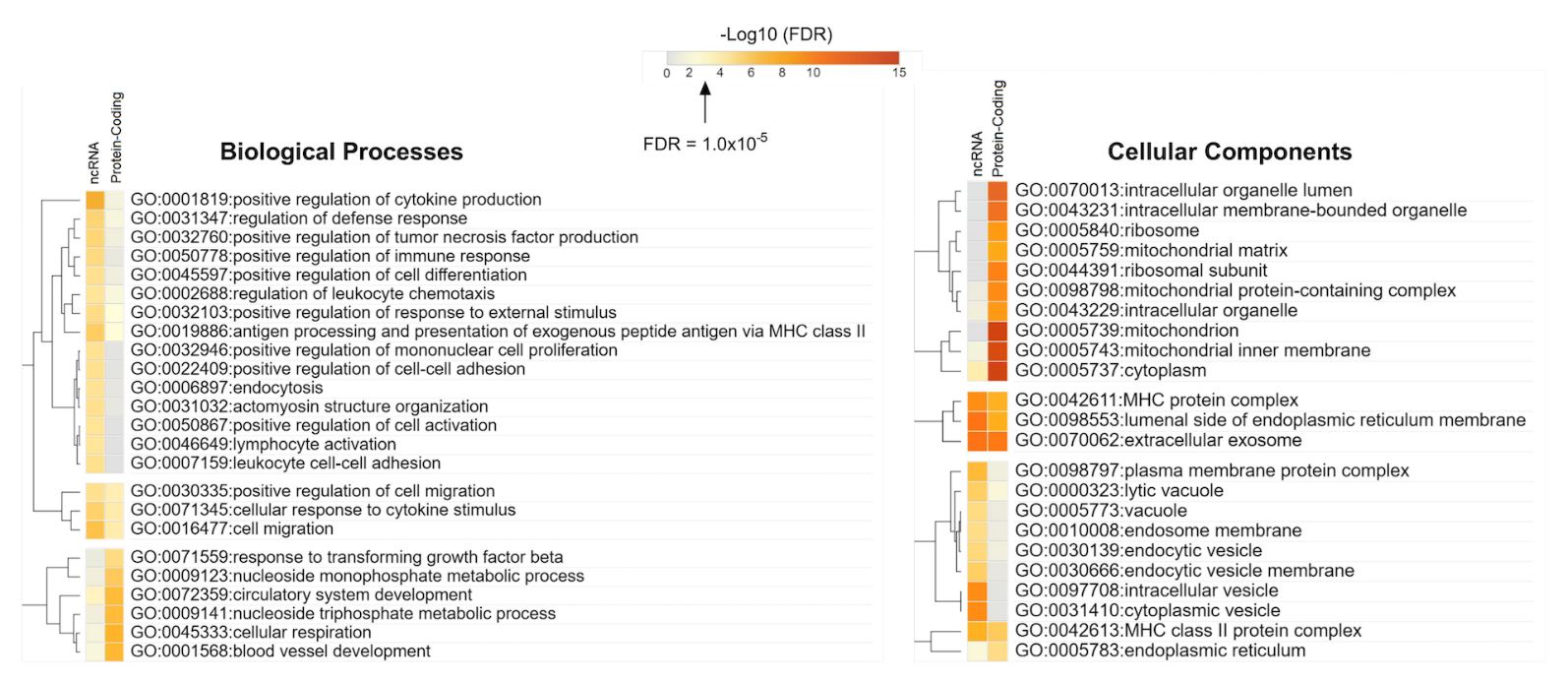
**

**Figure S7.** Similarities and differences in significant GO terms (biological processes and cellular components) across ncRNA MEGENA networks and our previously reported growth-regulated protein-coding MEGENA networks (5). The heatplot was created using Morpheus (https://clue.io/Morpheus), and genes and tissue types were hierarchically clustered using Euclidean distance (linkage method: complete).

References:

1. Morton,R.W. (2019) Resistance exercise-induced muscle hypertrophy.

2. Morton,R.W., Oikawa,S.Y., Wavell,C.G., Mazara,N., McGlory,C., Quadrilatero,J., Baechler,B.L., Baker,S.K. and Phillips,S.M. (2016) Neither load nor systemic hormones determine resistance training-mediated hypertrophy or strength gains in resistance-trained young men. *J Appl Physiol*, **121**, 129–138.

3. Phillips,B.E., Kelly,B.M., Lilja,M., Ponce-González,J.G., Brogan,R.J., Morris,D.L., Gustafsson,T., Kraus,W.E., Atherton,P.J., Vollaard,N.B.J., *et al.* (2017) A practical and time-efficient high-intensity interval training program modifies cardio-metabolic risk factors in adults with risk factors for type II diabetes. *Front Endocrinol (Lausanne)*, **8**, 1–11.

4. Mitchell,C.J., Churchward-Venne,T.A., Parise,G., Bellamy,L., Baker,S.K., Smith,K., Atherton,P.J. and Phillips,S.M. (2014) Acute post-exercise myofibrillar protein synthesis is not correlated with resistance training-induced muscle hypertrophy in young men. *PLoS One*, **9**, 1–7.

5. Stokes,T., Timmons,J.A., Crossland,H., Tripp,T.R., Murphy,K., McGlory,C., Mitchell,C.J., Oikawa,S.Y., Morton,R.W., Phillips,B.E., *et al.* (2020) Molecular Transducers of Human Skeletal Muscle Remodeling under Different Loading States. *Cell Rep*, **32**, 107980.

6. Wohlwend,M., Laurila,P.-P., Williams,K., Romani,M., Lima,T., Pattawaran,P., Benegiamo,G., Salonen,M., Schneider,B.L., Lahti,J., *et al.* (2021) The exercise-induced long noncoding RNA CYTOR promotes fast-twitch myogenesis in aging. *Sci. Transl. Med*, **13**, 7367.

7. Chang,M.-W., Yang,J.-H., Tsitsipatis,D., Yang,X., Martindale,J.L., Munk,R., Pandey,P.R., Banskota,N., Romero,B., Batish,M., *et al.* (2022) Enhanced myogenesis through lncFAM-mediated recruitment of HNRNPL to the MYBPC2 promoter. *Nucleic Acids Res*, **50**, 13026–13044.

8. Dill,T.L., Carroll,A., Pinheiro,A., Gao,J. and Naya,F.J. (2021) The long noncoding RNA Meg3 regulates myoblast plasticity and muscle regeneration through epithelial-mesenchymal transition. *Development (Cambridge)*, **148**.

9. Timmons,J.A., Knudsen,S., Rankinen,T., Koch,L.G., Sarzynski,M., Jensen,T., Keller,P., Scheele,C., Vollaard,N.B.J., Nielsen,S., *et al.* (2010) Using molecular classification to predict gains in maximal aerobic capacity following endurance exercise training in humans. *J Appl Physiol*, **108**, 1487–1496.

10. Liang,R., Shen,X., Wang,F., Wang,X., DesJarlais,A., Syed,A., Saba,R., Tan,Z., Yu,F., Ji,X., *et al.* (2021) H19X-encoded miR-322(424)/miR-503 regulates muscle mass by targeting translation initiation factors. *J Cachexia Sarcopenia Muscle*, **12**, 2174–2186.

11. Yue,Y., Yue,Y., Fan,Z., Meng,Y., Wen,C., An,Y., Yao,Y. and Li,X. (2023) The long noncoding RNA lnc-H19 is important for endurance exercise by maintaining slow muscle fiber types. *Journal of Biological Chemistry*, **299**, 105281.

12. Zhang,Y., Li,Y., Hu,Q., Xi,Y., Xing,Z., Zhang,Z., Huang,L., Wu,J., Liang,K., Nguyen,T.K., *et al.* (2020) The lncRNA H19 alleviates muscular dystrophy by stabilizing dystrophin. *Nat Cell Biol*, **22**, 1332–1345.
